# Oil contamination of sediments by freeze-drying versus air-drying for organic geochemical analysis

**DOI:** 10.1007/s10653-023-01594-9

**Published:** 2023-05-05

**Authors:** Chunqing Jiang, Rachel Robinson, Richard Vandenberg, Marina Milovic, Lisa Neville

**Affiliations:** Geological Survey of Canada, 3303-33 Street, NW, Calgary, AB T2L 2A7 Canada

**Keywords:** Freeze-drying, Air-drying, Sediments, Rock–Eval S1 peak, Contamination, Labile OM, UCM hump, Romulus lake

## Abstract

Freeze-drying is widely used in geochemical laboratories for preparing wet solid environmental samples such as sediments and soils before being analyzed for their contents and states of various metal elements and labile organic components that may be temperature- and/or redox-sensitive. Screening bulk geochemical analysis of two Artic lake sediment samples prepared by freeze-drying displayed unexpectedly high contents of labile organic matter (OM) represented by the Rock–Eval S1 peaks (e.g., 8.12 and 4.84 mg HC/g sediment). The amount of labile OM was reduced greatly for the freeze-dried sediment samples after a thorough cleaning of the freeze-drier sample chamber (e.g., 2.75 and 1.46 mg HC/g sediment), but was still significantly higher than that of the equivalent air-dried samples (e.g., 0.76 and 0.23 mg HC/g sediment). Compositional analysis of the labile OM fractions by gas chromatography (GC) of both freeze-dried and air-dried aliquots of the same sediments indicates the presence of unresolved complex mixture (UCM) humps of C_10_–C_23_ hydrocarbons in the freeze-dried samples. In contrast, air-dried samples, either real sediments or blank laboratory materials represented by clean sand and thermally spent shale, do not show the C_10_–C_23_ hydrocarbon UCM humps on their GC traces. The hydrocarbon UCM humps persist in the freeze-dried samples even they further went through air-drying at ambient conditions. Both bulk and compositional analytical results in this work appear to indicate the potential risk of introduction of external hydrocarbons to the prepared materials during freeze-drying process, especially if an aged freeze-drier was used without being thoroughly cleaned and if pump oil and cooling fluids were components of the device.

**Supplementary Information:**

The online version contains supplementary material available at 10.1007/s10653-023-01594-9.

## Introduction

Freeze-drying, also known as lyophilization or cryodesiccation, is a low temperature water removal process that involves freezing the target materials in a contained space at a temperature as low as -30 to -80 °C, lowering the pressure close to vacuum and then removing the water in the form of ice via sublimation. Thanks to its capability of better preserving temperature- and/or air-sensitive chemical components than traditional oven-drying and air-drying, the technique has been widely used in food processing (Chan et al., 2009; Fellows, 2009; Liapis & Bruttini, 2014; Ratti, 2001) and pharmaceutical manufacturing (Liapis & Bruttini, 2014; Nail et al., 2002; Nireesha et al., 2013) for better product quality. The technique has also been widely used in biological and medical laboratories for the preparation and preservation of biological tissue and materials for transport and storage (Abascal et al., 2005; Kodamatani et al., 2017; Liapis & Bruttini, 2014; Liu et al., 2009; Morgan et al., 2006; Qian & Zhang, 2011).

Marine, lake and river sediments as well as wet soil and peat samples used in environmental studies often need to be dried before solvent extraction for the analysis of organics, prior to acid digestion for instrumental analysis of major elements and trace metals, or before direct instrumental examination such as thermal spectroscopy and XRD analyses. Owning to its effectiveness in minimizing heat-associated loss of volatile organics and air-related degradation of redox-sensitive elements as well as efficiency in removing water, freeze-drying has also been extensively employed in environmental laboratories for pre-treatment of water-containing solid samples (Beriro et al., 2014; Geffard et al., 2004; Kersten & Förstner, 1987; Kodamatani et al., 2017; Liu & Lee, 2006; McClymont et al., 2007; Pérez-Fernández et al., 2015; Simpson et al., 2019; Söderström et al., 2005; Zhang & Scherer, 2011). For example, a study of biomarkers in ODP cores by McClymont et al. (2007) showed that air-drying can incur significant losses of chlorins (> 25%) and alkenones (up to 75%) compared with freeze-drying. Quantitative analysis of polycyclic aromatic hydrocarbons (PAHs) in a homogenized gasworks soil sample by Beriro et al. (2014) showed that the concentrations of low molecular weight PAHs such as naphthalenes are significantly higher in freeze-dried and air-dried samples than in the oven-dried samples. Simpson et al. (2019) found that the differences in phosphate concentration between fresh and freeze-dried sediments were smaller and more consistent than between fresh and air-dried sediments.

Among the various organic and inorganic geochemical characteristics, the bulk properties of organic matter (OM) in the dried matrices revealed via Rock–Eval analysis has been one focus of many recent studies (Baudin et al., 2015; Deison et al., 2012; Galloway et al., 2018; Newell et al., 2016; Outridge et al., 2007; Sanei & Goodarzi, 2006). Through sequential stages of programmed pyrolysis and combustion, volatile to semi-volatile OM, reactive solid OM and refractory solid OM in a solid sample can be quantitatively determined from Rock–Eval analysis as a function of temperature and are generally expressed as S1 peak, S2 peak, productive organic carbon (PC), residual or refractory organic carbon (RC) and total organic carbon (TOC). The variation of different states of OM in sediments has been used to illustrate the evolution of paleo-climate, paleo-hydrogeology and paleo-vegetation as well as the accumulation and distribution of some metal elements throughout deposition profiles. Together with hydrocarbon compositions from gas chromatography (GC) analysis of the soluble OM fractions, bulk OM properties from Rock–Eval analysis of freeze-dried Holocene peat core samples were used by Newell et al. (2016) to reveal the wetness variation and groundwater fluctuation during their 4000 years of deposition in the Lambourn Valley of Southern England. Deison et al. (2012) reported that the concentrations of methyl mercury in sediments collected from different lakes across the Mackenzie Delta uplands of NW Canada showed positive correlation (*r*^2^ of 0.71–0.79) with the Rock–Eval TOC contents and S1 and S2 peak values of the freeze-dried samples. Wu et al. (2013) reported statistically significant correlation between Rock–Eval S1 values and the concentrations of Hg measured on freeze-dried sediment samples from two Chinese lakes and thus concluded a controlling role of soluble organic matter on the accumulation of Hg in sediments. Similar conclusions had been made previously on recent lake sediments from Alberta, Canada, by Sanei and Goodarzi (2006) and on Canadian High Arctic lake sediments by Outridge et al. (2007). A recent study on the distribution of arsenic (As) in lake sediments from subarctic Canada by Galloway et al. (2018) also reported that labile organic matter represented by Rock–Eval S1 peaks are significantly related to the concentrations of sedimentary As and sulfur.

The significant correlations of Rock–Eval S1 peaks with the concentrations of those environmentally toxic and societally conscientious metals in recent sediments found by previous studies underline the importance of an accurate understanding of the composition of S1 peaks from Rock–Eval analysis. Employing thermal desorption–gas chromatography (TD–GC) and solvent extraction followed by gas chromatography mass spectrometry (GC–MS) analyses, this study aims to investigate the potential of hydrocarbon contamination to sediment samples from freeze-drying that may lead to misleadingly high S1 peaks by Rock–Eval analysis.

## Samples and methods

### Samples

A total of five solid matrix samples have been used in this study (Table 1). Two of them are natural sediment samples collected wet from Romulus Lake (79.8919° latitude, −84.5500° longitude) and Twin Lakes (79.8422° latitude, −87.5308° longitude), respectively, in the Canadian High Arctic. The sediment samples had been kept in Ziploc bags and stored frozen throughout transportation and storage before pre-treatment in the laboratories for further analyses. The third sample, termed “RE standard shale,” is a powdered shale sample used as a QC/QA standard for Rock–Eval analysis at the Geological Survey of Canada, Calgary (GSC-Calgary). The fourth sample, termed “RE spent shale,” is a homogenized composite of residual shaly rock samples after routine Rock–Eval analyses that first underwent pyrolysis from 300 to 650 °C and then combustion from 300 to 850 °C as specified in the Rock–Eval basic method (Behar et al., 2001; Lafargue et al., 1998). The OM content of the RE spent shale is close to zero considering the extreme thermal conditions it had been exposed to. The fifth sample is a commercially acquired Ottawa sand that has been used in GSC-Calgary laboratories (and many other laboratories as well) for cleaning grinding devices and as blank media for open column chromatography separation. The later three laboratory rock/solid samples were mixed with deionized water in the proportion of 20 g per 15 mL in the study to mimic water-laden wet samples requiring drying treatment.

**Table 1:** Results of Rock–Eval analysis, solvent extraction and open column chromatography separation for the freeze-dried and air-dried lake sediment and laboratory rock samples used in this study

Sample	Drying method		S1	S2	S3	S4	Tmax (°C)	TOC (%)	MinC (%)	Extra Yield (%)	SARA composition (%)		
			(mg HC/g rock)		(mg CO_2_/g rock)						Saturate	Aromatic	Polars
Romulas lake sediment	Freeze-dried Air-dried		2.75 0.76	10.81 8.94	7.75 7.71	86.20 92.33	422 425	4.16 4.05	0.28 0.29	1.42 0.21	52.81 11.60	28.19 17.31	19.00 71.09
Twin lakes sediment	Freeze-dried Air-dried		1.46 0.23	2.47 2.20	4.03 4.58	55.33 57.18	391 403	2.22 2.17	0.21 0.20	0.72 0.05	60.61 32.56	28.90 11.63	10.49 55.81
RE standard shale*	Freeze-dried Air-dried		0.31 0.06	1.74 1.31	1.36 1.38	70.79 67.56	410 411	2.71 2.53	0.18 0.16	0.27 0.02	63.20 29.94	26.51 9.26	10.29 60.80
RE spent shale*	Freeze-dried Air-dried		1.68 0.02	0.33 0.08	0.42 0.35	1.94 0.69	n.a n.a	0.25 0.05	1.58 1.39	0.16 0.00	64.94 n.a	21.87 n.a	13.19 n.a
Ottawa sand*	Freeze-dried Air-dried		0.01 0.00	0.02 0.01	0.07 0.06	0.03 0.06	n.a n.a	0.00 0.00	0.01 0.01	0.00 0.00	n.a n.a	n.a n.a	n.a n.a
RE spent shale*	Freeze-dried	10 cm OD petri dish 7 mm id crucible	1.71 0.14	0.27 0.13	0.43 0.41	2.07 1.82	n.a n.a	0.26 0.11	0.86 0.52	0.22 0.14	62.23 68.41	30.11 24.93	7.66 6.65

^*^Mixed with water in the proportion of 20 g/15 mL; n.a.: not applicable; SARA: saturate–aromatics–resin–asphaltene by open column chromatography separation

### Sample drying

In order to investigate the potential effect of hydrocarbon contamination from freeze-drying, aliquots of the frozen sediment samples and water-laden laboratory samples were dried by both freeze-drying and air-drying. The two frozen sediment samples were each thawed and mixed well before being divided into two aliquots, respectively. One aliquot of each wet sediment sample was treated by freeze-drying using a Labconco Freezon® Plus 12 freeze-drier. The vacuum freeze-drier was operated at −35 °C for 3 days. The 2nd aliquots of the sediment samples were air-dried by being left in a fume hood at ambient conditions for 72 h. The laboratory rock samples, RE standard shale, RE spent shale and Ottawa sand mixed with deionized water were also divided into halves, with one-half for 3-day freeze-drying and the other half for 3-day air-drying under the same conditions as for the lake sediments. Samples were spread out on petri dishes (10 cm OD) for both freeze-drying and air-drying to allow maximum sample exposure to the vacuum or air but with the petri dishes covered with a piece of Kimwipes tissue. In addition, an experiment to examine the effect of exposed surface area on the contents of OM in freeze-dried sediments was also conducted by comparing freeze-drying aliquots of the wet RE spent shale spread out on a 10 cm OD petri dish versus in a 7 mm ID Rock–Eval sample crucible under the same conditions (Table 1).

Both the freeze-dried and air-dried subsamples were promptly powdered using a mortar and pestle to homogenize and stored in sealed vials. Aliquots of the powdered dried subsamples were then subjected to (1) Rock–Eval analysis for bulk OM properties; (2) TD–GC analysis for fingerprinting molecular compositions of the volatile and semi-volatile OM equivalent to Rock–Eval S1 peaks; and (3) solvent extraction followed by full scan GC–MS analysis of the whole extracts.

### Rock–Eval analysis

Rock–Eval analysis was carried out in duplicates or triplicates on a Vinci Technologies’ Rock–Eval 6 Turbo device with dual pyrolysis and combustion capabilities following the *Basic Method* as described by Behar et al. (2001), and details of the procedure were given in Jiang et al. (2017). Briefly, aliquots of powdered dried samples in appropriate amounts (*ca*. 40 to 55 mg for lake sediment, ~ 70 mg for RE spent shale and 90–100 mg for laboratory sand) were used for the analysis in this study. Initially the samples were heated at 300 °C in an inert atmosphere (i.e., N_2_) for 3 min to produce the S1 peaks representing labile OM. The temperature of the pyrolysis furnace was then ramped up from 300 to 650 °C at a rate of 25 °C/minute, yielding an S2 peak that represents thermal decomposition products from the nonvolatile reactive OM. CO_2_ and CO released from OM before 390 °C during the pyrolysis stage were recorded as S3 peaks as part of productive organic OM (or PC) in addition to S1 and S2 peaks. The temperature at the maximum of S2 peak varies with the thermal/burial history of the sedimentary OM and is typically converted to Tmax (°C), the widely accepted thermal maturity parameter. Following pyrolysis, samples were transferred to the combustion furnace of the instrument where they were linearly heated from 300 to 850 °C under airflow to oxidize the RC to form S4 CO_2_ and CO peaks and then decompose the rest of carbonate minerals to S5 CO and CO_2_ peaks. The TOC is the sum of the PC and RC, and similarly, mineral carbon (MinC) is the sum of the pyrolysis and oxidation mineral carbon. Frequent analysis of a powdered shale rock sample from the Cretaceous 2nd White Specks Formation as Rock–Eval standard has been performed at the GSC-Calgary for QC/QA purpose to ensure data consistency and accuracy.

### TD–GC analysis

TD–GC analysis using a Frontier EGA/PY-3030D pyrolyzer coupled to an Agilent GC–MSD/FID dual detection system has been used in this study to characterize the composition of volatile and semi-volatile OM in the dried solid samples corresponding to Rock–Eval S1 peaks. Details of the TD–GC–MS/FID analysis can be found in Jiang et al. (2016) and following is a brief description of the method.

Aliquots of powdered dried samples in the amounts similar to Rock–Eval analysis were used for the TD–GC–MS/FID analysis. Thermal desorption in the EGA/PY-3030D pyrolyzer was carried out at 300 °C for 3 min to duplicate the release of labile OM equivalent to Rock–Eval S1 peaks. Upon the commencement of thermal desorption in the pyrolyzer, the OM thermally released from the solid samples were immediately carried away from the pyrolyzer by a flow of helium carrier gas and then cryo-trapped at − 186 °C at the head of the capillary column of the GC–MSD/FID system. The GC–MS/FID analysis was initiated immediately after the 3-min thermal desorption was completed, with the cryo-trapping section of the column being heated at a rate of 800 °C/min to release the cryo-trapped OM into the GC column for molecular level separation. The capillary column used for the GC analysis was a PONA 50 m × 0.20 mm × 0.5 μm, and helium was used as carrier gas at a constant flow rate of 1 mL/min. The GC oven temperature was initially held at 33 °C for 10 min, followed by an increase at 3 °C/min to 63 °C, and finally increased at 6 °C/min to 325 °C and maintained for 35 min. The flow from the GC column was split between MSD and FID detectors for dual detection, with the former being operated in full scan mode for compound identification and the latter for quantification. Compound identification was based on comparison of GC retention times and mass spectra with those in literature as well as data collected historically at GSC-Calgary.

### GC–MS analysis of solvent extracts and saturated fractions

5–20 g of each powdered sample was subjected to Soxhlet extraction for 72 h using dichloromethane (DCM) as solvent. Activated copper grains were added to the extract contents at the end of extraction to remove any elemental sulfur. After removing most of the solvent using a rotary evaporator and filtration to remove any solids, the extract–solvent contents were treated with a gentle stream of nitrogen to reach a concentration level appropriate for full scan GC–MS analysis. After satisfactory GC–MS analysis, further solvent removal by gentle nitrogen blow was performed to obtain the weights of extracts.

GC–MS analysis of the whole extracts was carried out on an Agilent 6890 GC interfaced to a 5973 mass selective detector (MSD). An Agilent 30 m × 0.32 mm × 0.25 μm DB-5ms column was used for the GC with helium as carrier at a flow rate of 1 mL/min. The GC injector was set at 300 °C with a 40:1 split injection being employed. The GC oven temperature was initially set at 40 °C, programed at 4 °C/min to 325 °C and then held for 15 min. The MSD was operated in full scan mode over the range of *m/z* 50−550. Compound identification of the GC–MS peaks was achieved by comparing the GC retention times and mass spectra with those published in literature and/or available in the NIST library.

## Results and discussions

### Bulk OM properties from Rock–Eval analysis and solvent extraction

To determine the contents of various types (e.g., labile, reactive solid and refractory solid) of OM in sediments from the two Arctic lakes, aliquots of the two waterlogged lake sediment samples were prepared initially by both freeze-drying and air-drying for comparison. Results of subsequent Rock–Eval analyses are presented in Table S1 and Figure S1 showing that, compared with the (ambient) air-dried sediment equivalents, the freeze-dried sediment samples display much higher S1 peaks (8.12 vs. 0.62 and 4.84 vs. 0.31 mg HC/g sediment, respectively), moderately enhanced S2 peaks (14.24 vs. 8.71 and 4.48 vs. 2.73 mg HC/g sediment, respectively). Correspondingly, the measured TOC values are also higher for the freeze-dried than the air-dried sediment samples (e.g., 5.54% vs. 3.80% and 3.39% vs. 2.44%, respectively). Subsequently, a thorough cleaning of the freeze-drier sample chamber was performed using DCM-wetted paper towel and Kimwipes tissues before the wet sediment and waterlogged solid laboratory samples were freeze-dried again for bulk and compositional characterization of their OM.

**Fig. 1:**
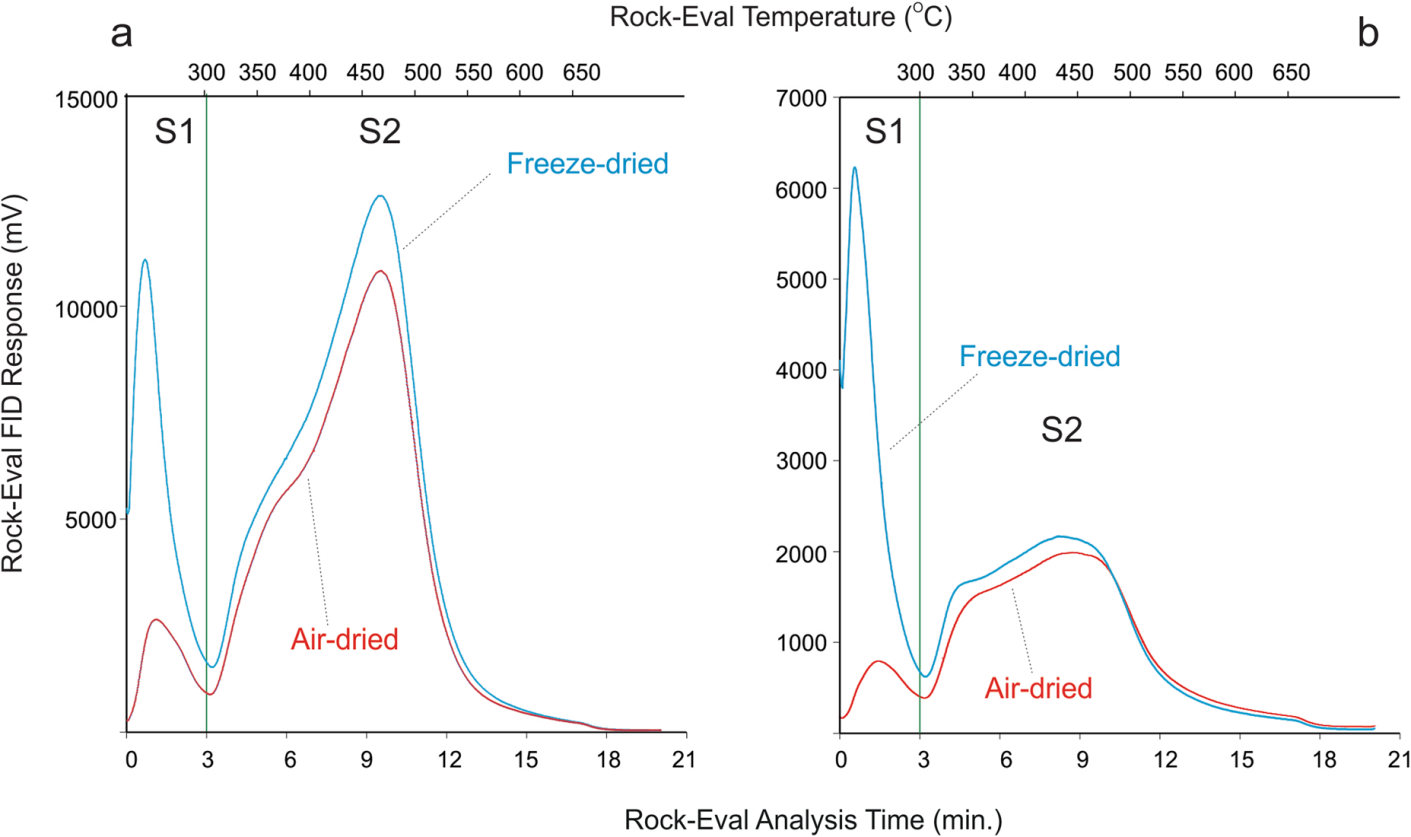
FID hydrocarbon pyrograms from Rock–Eval pyrolysis of freeze-dried (blue line) vs. air-dried (red line) sediment samples from **a** Romulus Lake and **b** Twin Lakes from the Canadian High Arctic

Table 1 presents major Rock–Eval and solvent extraction results on the subsamples prepared from the two lake sediment and the three laboratory samples by freeze-drying (after device maintenance) and air-drying, respectively. As anticipated, the Ottawa sand subsamples, either freeze-dried or air-dried, contain little amount of any types of OM, and the detected values are well within the accuracy of the bulk thermal analytical technique. In contrast, a pronounced and consistent difference in the contents of different types of OM exists between the freeze-dried and air-dried subsamples of the two lake sediments, the RE standard shale and the RE spent shale. For the shaly matrix samples, the freeze-dried subsamples all display a much larger S1 peak (in mg HC/g solid) than the corresponding air-dried subsamples (e.g., 2.75 vs. 0.76 for the Romulus lake sediment; 1.46 vs. 0.23 for the Twin Lakes sediment; 0.31 vs. 0.06 for the RE standard shale; and 1.68 vs. 0.02 for the RE spent shale). The freeze-dried subsamples also show consistently higher S2 values than their air-dried counterparts (Table 1). The contrast between the freeze-dried and air-dried subsamples in their S1 and S2 peaks is graphically represented in Fig. 1 showing the hydrocarbon pyrograms from Rock–Eval analysis of the two lake sediments. It is also apparent that the increased S2 contents in the freeze-dried subsamples seem to mainly occur as front shoulders to the S2 peaks (Fig. 1). This is similar to Rock–Eval results on petroleum source rock samples that contain heavy hydrocarbons and bitumen due to lower maturity or are contaminated by drilling additives (Abrams et al., 2017; Jiang et al., 2019; Sanei et al., 2020).

The above observations based on Rock–Eval analytical results are actually also supported by results from solvent extraction that produced significantly higher amounts of extractable OM on the freeze-dried than the air-dried lake sediment subsamples (Table 1). In addition, the extractable OM from the freeze-dried subsamples have much higher contents (wt%) of hydrocarbons than in the air-dried samples. Accordingly, the shaly lake sediment subsamples prepared via freeze-drying all exhibit a higher TOC content than those prepared by air-drying.

That an elevated amount of OM especially the labile OM represented by S1 peaks and solvent extractable OM has been detected in the freeze-dried compared with the air-dried subsamples seems to indicate an introduction of volatile and semi-volatile OM to the samples during freeze-drying as a result of contamination. This is further supported by the fact that significant S1 peaks were also detected in the freeze-dried RE spent shale that originally contained no volatile OM at all due to extreme thermal treatment (Table 1). There is a possibility that evaporative loss of volatile components occurred to the air-dried subsamples leading to reduced S1 peaks, but this cannot fully account for their sharp difference from the freeze-dried counterparts.

Despite the variation in the quantity of various phases of OM between freeze-dried and air-dried subsamples, subsamples prepared by the two different drying methods have consistent Rock–Eval *T*max and Min*C* values, indicating that the contamination from freeze-drying has not significantly affected the maturity- and carbonate-related parameters that are mostly independent of the presence of labile OM.

### Molecular compositions of labile OM by TD–GC and solvent extract GC–MS analyses

**Fig. 2:**
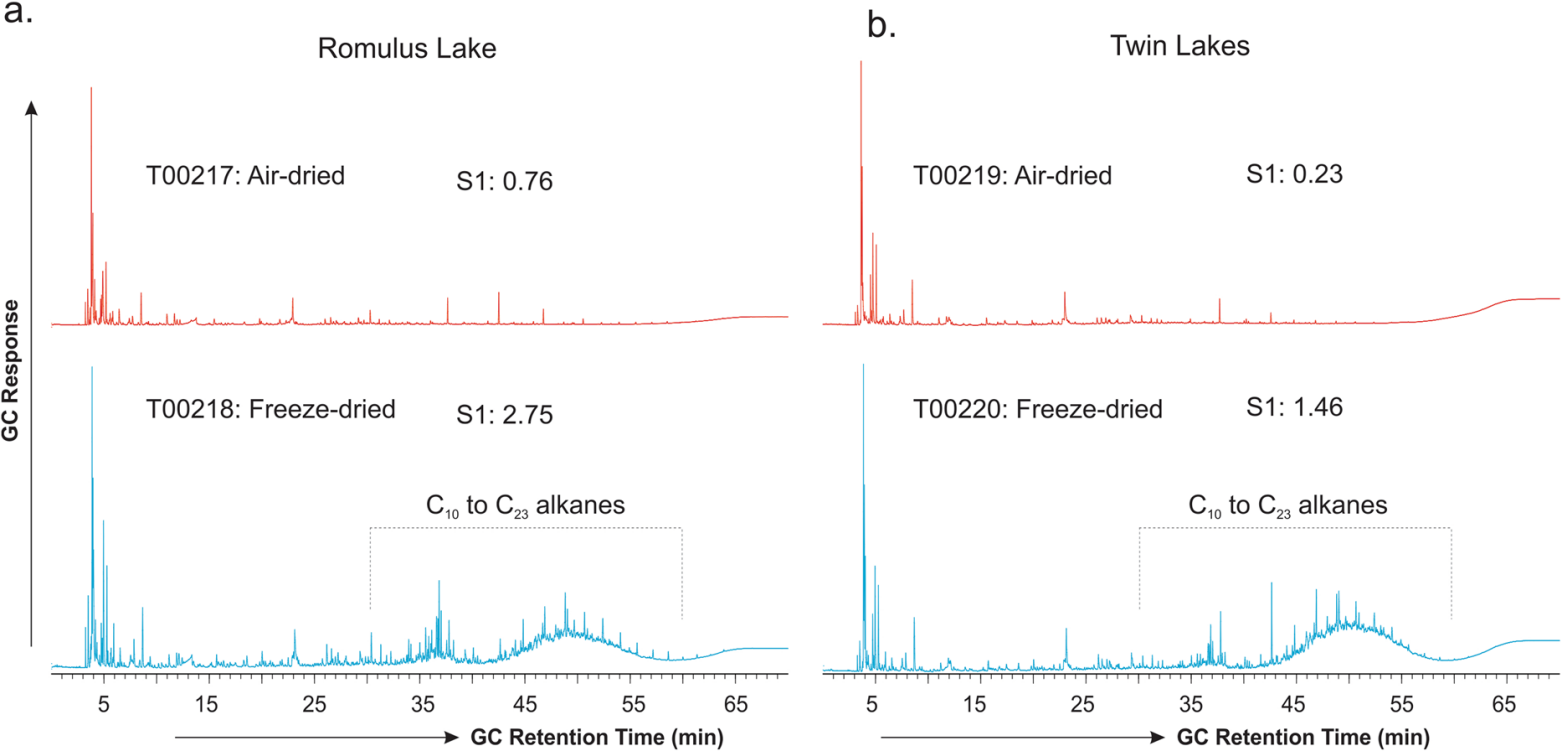
TD–GC traces showing the molecular compositions of Rock–Eval S1 equivalent volatile and semi-volatile hydrocarbons in the freeze-dried vs. air-dried sediment samples from **a** Romulus Lake and **b** Twin Lakes from the Canadian High Arctic. Note that GC response has been normalized to the weight of samples

Figure 2 displays the TD–GC traces for the two sediment samples from Romulus Lake and Twin Lakes after freeze-drying and air-drying, respectively, showing the compositions of their S1-equivalent hydrocarbons. Both the freeze-dried and air-dried subsamples show similar hydrocarbon compositions in the < C_10_ range for each sediment, with the major peaks in the front likely being the thermal decomposition products (e.g., ethylene oxide, hydroxyacetic acid, methyl propene, acetone and furans based on their mass spectra) of certain reactive sedimentary OM. Nevertheless, distinction is obvious as well in the compositions of thermally labile OM. Compared with the air-dried subsamples (red in Fig. 2), the freeze-dried sediment samples (blue in Fig. 2) exhibit pronounced unresolved complex mixture (UCM) humps in the range of C_10_–C_23_ hydrocarbons with recognizable *n*-alkanes and isoprenoids pristane and phytane seated atop.

**Fig. 3:**
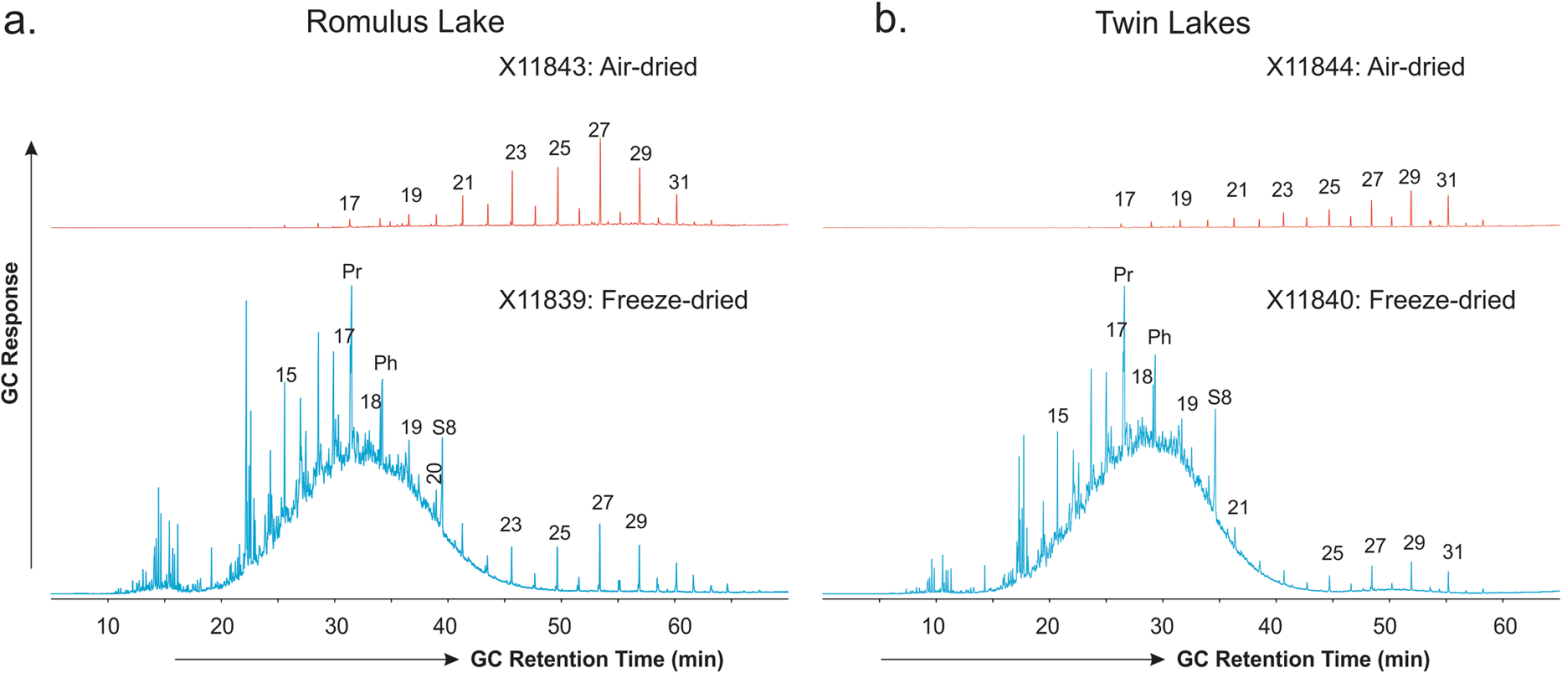
TIC traces from full scan GC–MS analysis of saturated fractions of solvent extracts from the freeze-dried vs. air-dried sediment samples from **a** Romulus Lake and **b** Twin Lakes in the Canadian High Arctic. Note that GC response has been normalized to the weight of samples. Numbers atop the peaks represent the carbon numbers of the corresponding n-alkanes; Pr: pristane; Ph: phytane

The unexpected occurrence of the C_10_–C_23_ UCM hydrocarbon components in the freeze-dried relative to the air-dried subsamples revealed by TD–GC analysis is also manifested by the GC analysis of the whole solvent extracts and the saturated fractions of the solvent extracts. Figure 3 shows the total ion chromatogram (TIC) traces from full scan GC–MS analysis of whole solvent extracts of the two lake sediment samples prepared by both freeze-drying and air-drying. The air-dried sediment samples show a hydrocarbon composition dominated by C_21_–C_33_ *n*-alkanes with odd-over-even preference (Fig. 3), a feature typical of terrestrial plant material (Peters et al., 2004). In contrast, the C_10_–C_23_ UCM humps predominate over the terrestrial-sourced C_21_–C_33_ *n*-alkanes in the extracts of the freeze-dried sediment samples.

**Fig. 4:**
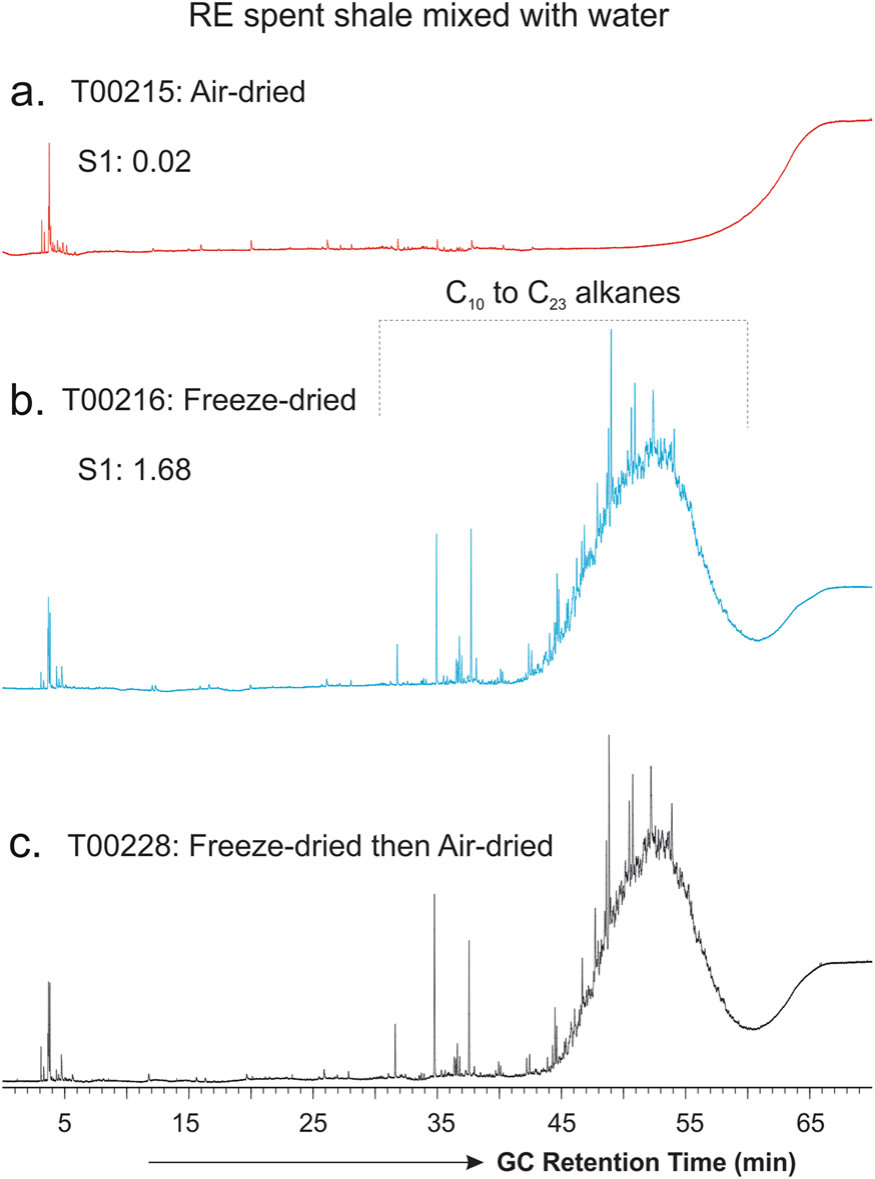
TD–GC traces showing the molecular compositions of Rock–Eval S1 equivalent volatile and semi-volatile hydrocarbons in the RE spent shale after being **a** air-dried; **b** freeze-dried for 48 h; and **c** freeze-dried followed by air-drying for 48 h. Note that GC response has been normalized to the weight of samples

Together with the bulk extraction yield and SARA composition (Table 1), compositional analyses of the lake sediment samples further indicate a significant introduction of external hydrocarbons to the sediments during freeze-drying compared with air-drying. This is further demonstrated by the TD–GC analysis of the RE spent shale samples (Fig. 4). The air-dried RE spent shale shows little hydrocarbon components released from thermal desorption at 300 °C except a few minor peaks in the front (Fig. 4a), and this, as anticipated, is because the shale material has previously been exposed to as high as 850 °C thermal treatment through Rock–Eval analyses. Contrary to this, the freeze-dried RE spent shale displays a TD–GC trace dominated by C_10_–C_23_ hydrocarbon UCM hump (Fig. 4b), similar to that of the freeze-dried lake sediments except the absence of higher-plant derived C_21_–C_33_ *n*-alkanes (blue in Fig. 2). The intensity and distribution pattern of the TD–GC trace (e.g., UCM hydrocarbon hump) of the freeze-dried RE spent shale show little variation even after the freeze-dried shale material was further air-dried for 48 h (Fig. 4c). This suggests that the UCM hydrocarbons seem to have been stably absorbed onto the shale matrices and that 48 h of air-drying at ambient conditions did not cause significant desorption and loss of these hydrocarbons from the shale material.

### Freeze-drying as a potential source of hydrocarbon contamination

**Fig. 5:**
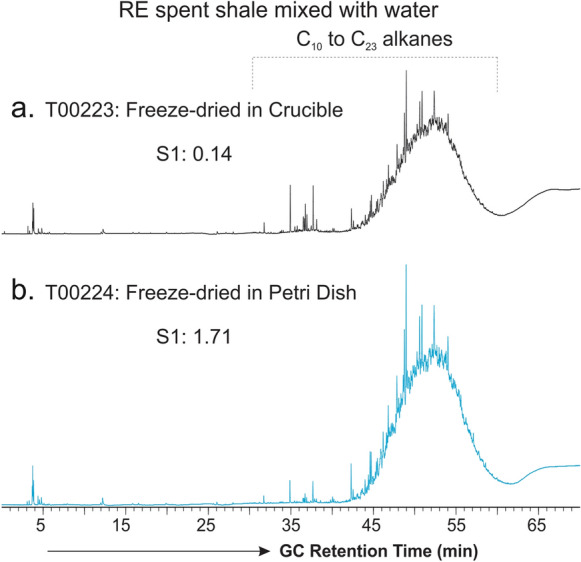
TD–GC traces showing the molecular compositions of Rock–Eval S1 equivalent volatile and semi-volatile hydrocarbons in the RE spent shale samples **a** freeze-dried in a 7 mm ID and 10 mm deep crucible; and **b** freeze-dried by spreading on a petri dish, respective for 48 h. Note that GC response has been normalized to the weight of samples

The results presented above clearly indicate that freeze-drying can potentially cause hydrocarbon contamination to the samples. The freeze-drier used in this study is a Labconco Freezon® Plus 12 that had been in use for over 20 years. Vacuum pump oil is utilized for the device to achieve the vacuum and heat transfer fluid Lexsol 542 used to achieve the low temperature required for the accelerated removal of water through ice sublimation. As the waterlogged solid samples to be dried were placed in petri dishes that were covered with laboratory grade Kimwipes tissues, the contaminant hydrocarbons seem to be likely picked up by the solid matrix via surface adsorption from the “vacuum” in the freezing chamber. This is supported by a comparison of the TD–GC results on two RE spent shale subsamples that were freeze-dried in the same batch and at the same weight loads, but with one placed in a 7 mm ID and 10 mm deep Rock–Eval crucible (Fig. 5a) and the other spread out on a 10 cm OD petri dish (Fig. 5b). Compared with the RE spent shale freeze-dried in a narrow and deep crucible, the RE spent shale sample freeze-dried on a petri dish had much larger surface exposure in the sample chamber. Consequently, the later shows more contaminated hydrocarbons than the former, evident from both the Rock–Eval S1 values and the relative intensities of the UCM humps on their TD–GC traces (Fig. 5b vs. 5a).

**Fig. 6:**
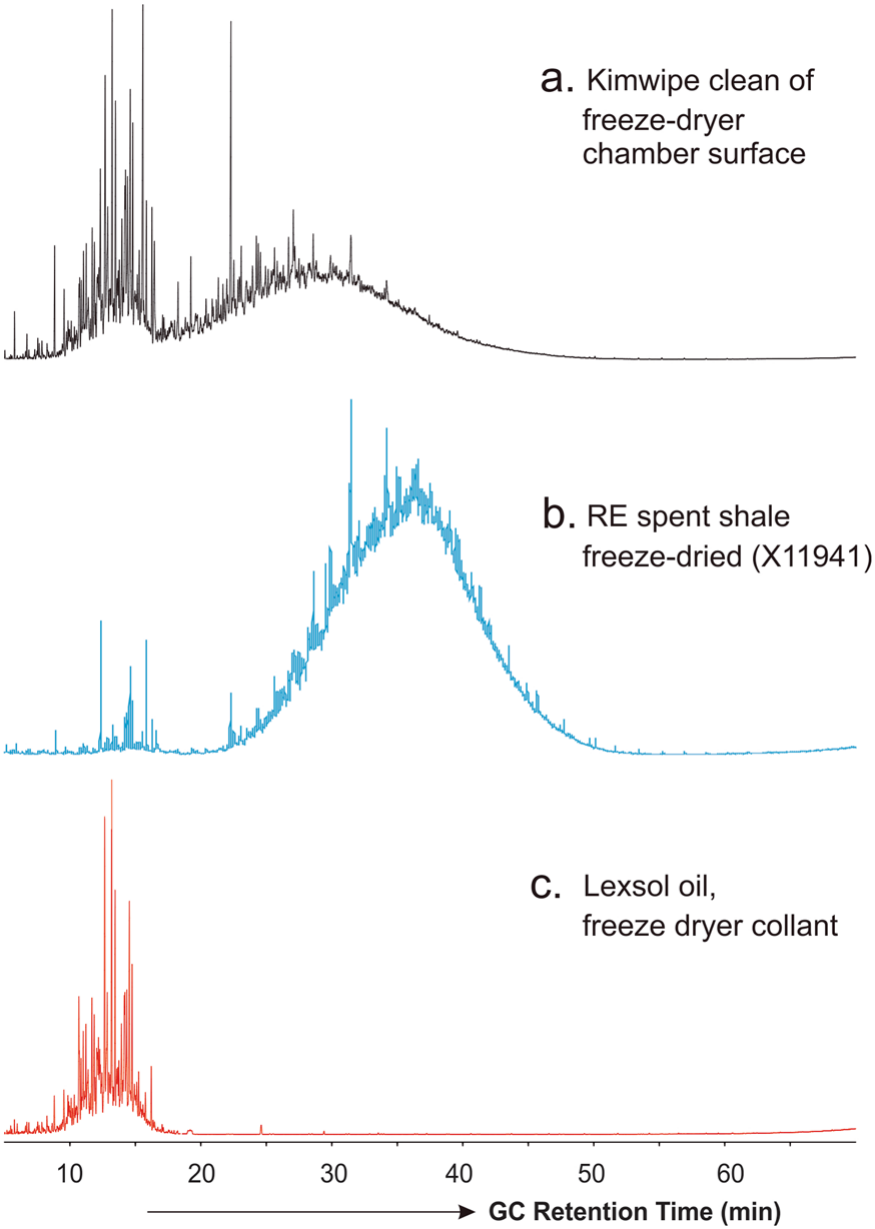
TIC traces from full scan GC–MS analysis of whole solvent extracts from **a** Kimwipes after wipe-cleaning freeze-drier chamber wall; **b** freeze-dried RE spent shale; and **c** heat transfer fluid Lexsol 542 used for the freeze-drying device

Following the finding of the contamination issue, thorough cleaning of the freeze-drying chamber was performed using Kimwipes wetted with DCM solvent. Figure 6 presents the TIC traces from full scan GC–MS analysis of the solvent rinsing content of the Kimwipes tissues used for cleaning, together with the TIC of extract from a freeze-dried RE spent shale sample. It is clear that the Kimwipes-cleaning content from the freeze-drier chamber (Fig. 6a) display a (17–50 min) UCM hydrocarbon hump similar to that of the solvent extracts of freeze-dried RE spent shale (Fig. 6b) and lake sediment samples (Fig. 3), indicating that the hydrocarbon contamination was indeed from the freeze-drying chamber. The gentler UCM hump in the front part (i.e., retention time 5–17 min) of the TIC trace of Kimwipes extract (Fig. 6a) is likely due to the vapor condensate from the heat transfer fluid Lexsol 542 used with the freeze-drier an example GC–MS TIC trace for which is presented in Fig. 6c.

Figure S2 shows the TD–GC traces for the freeze-dried vs. air-dried Ottawa sand samples. Similar to the Rock–Eval results of very low S1 and S2 peaks for both samples (Table 1), little hydrocarbon contamination was detected in both the freeze-dried and the air-dried clean sand samples by the compositional analyses. This is likely because, compared with the shaly sediment or rock samples, clean Ottawa sand has no clay content and thus much reduced adsorption capacity for hydrocarbon vapor in the freeze-drier chamber.

### *Implications for preparing wet sediment samples *via* freeze-drying*

Freeze-drying has been widely used in preparation of wet sediment samples for the determination of various environmentally sensitive organic components and heavy metal elements (Beriro et al., 2014; Galloway et al., 2018; Outridge et al., 2007; Sanei & Goodarzi, 2006; Söderström et al., 2005; Wu et al., 2013; Yang et al., 2018). Although those temperature- and/or redox-sensitive organics and heavy metals were not the target analytes in this study, Figure S3 clearly shows that freeze-drying does have better preservation of sediment sample against air-induced oxidation. The occurrence of elemental sulfur due to oxidation of sulfide was significantly reduced (by 95%) in the freeze-dried compared with the air-dried lake sediment samples (blue vs. red in Fig. S3a). This is consistent with the observation that fresh copper grains turned dark immediately when added to the solvent extraction contents from air-dried sediments but remained shiny for longer time in the extraction contents of freeze-dried sediments during post-extraction solvent removal. Nevertheless, the addition of excess fresh copper grains effectively removed the newly formed elemental sulfur from the extraction contents of both freeze-dried and air-dried sediments in this study (Fig. S3b).

Despite the better sample preservation by freeze-drying, caution and awareness should be raised among (environmental) scientists about the potential contamination from freeze-drying when preparing shaly sediment samples and its impact on our understanding of targeted environmental issues. Söderström et al. (2005) reported much elevated concentrations of polychlorinated biphenyls (PCB) in freeze-dried versus air-dried sediments (i.e., 189 vs. 7 ppb dry mass) from Swedish lakes, and they attributed that to the adsorption of PCB from the laboratory air during freeze-drying. While the introduction of exotic hydrocarbons from freeze-drying as reported in this study may not directly affect the concentrations of analytes such as pesticides, arsenic and mercury (had them been analyzed), such contamination may significant elevate the measured contents of labile OM (represented by S1) in shale sediments of low contents of OM. This can potentially mask an already-existing correlation between labile OM and other target analytes of interest, thus complicating our understanding of their sources and implications. The bulk organic properties from Rock–Eval analysis have been increasingly used for evaluating soil conditions (Derenne & Quéné, 2015; Gregorich et al., 2015; Marchand et al., 2008) and peat ecosystem (Delarue et al., 2013; Grice et al., 2007; Newell et al., 2016). In addition, the contents of thermally labile OM represented by the Rock–Eval S1 values in sediments from serval lake settings have been found to be significantly correlated to the concentrations of mercury (Outridge et al., 2007; Sanei & Goodarzi, 2006; Wu et al., 2013) and arsenic (Galloway et al., 2018). Findings presented above in this work clearly show that the efficacy of Rock–Eval S1 results on such applications could be potentially compromised by any oil/hydrocarbon contamination if an aged vacuum freeze-drier was used for sample drying preparation and if no molecular fingerprinting was performed to confirm the composition of S1-equivalent components and qualify the bulk analytical results. Therefore, diligent maintenance with thorough cleaning of sample chamber is highly recommend when an (aged) freeze-drier equipped with oil vacuum pump is used for preparing wet sediment and soil samples for the analysis of OM, especially labile OM.

## Conclusions

Lake sediment and laboratory shaly rock material samples prepared using an old freeze-drier were found to display much higher contents of labile OM than the corresponding samples prepared by air-drying in open air at ambient conditions. Compositional analyses of the labile OM via direct TD–GC of the prepared samples and their solvent extract GC indicate the occurrence of hydrocarbon contamination to the freeze-dried samples that was not detectable in the air-dried shaly samples. However, the hydrocarbon contamination was not detected in the freeze-dried clean sand sample, same as the air-dried sand. In addition, the extent of hydrocarbon contamination from freeze-drying appears to be related to the surface area of the samples being prepared: the larger exposure surface of the sample, the higher amount of hydrocarbon contamination from freeze-drying. The source of hydrocarbon contamination appears to be from the adsorption of vapor of vacuum pump oil and heat transfer fluid/oil utilized by for the freeze-drying device, suggesting that regular and frequent maintenance of a (aged) freeze-drier is necessary for quality preparation of sediment and other water-lodged solid samples. Despite the potential oil contamination issue, freeze-drying was found to provide better sample preparation against oxidation from ambient air-drying.

### Supplementary Information

Below is the link to the electronic supplementary material.

Supplementary file1 (DOCX 394 kb)
