# Supplementary material for: Oil contamination of sediments by freeze-drying versus air-drying for organic geochemical analysis
Source: Environ Geochem Health. 2023 May 5;45(8):5799–811. doi: 10.1007/s10653-023-01594-9 (PMC10403420; doi:10.1007/s10653-023-01594-9)
Supplement: Supplementary file 1 — Supplementary file1 (DOCX 394 kb) [file 10653_2023_1594_MOESM1_ESM.docx]

**Oil contamination of sediments by freeze-drying versus air-drying for organic geochemical analysis**

Chunqing Jiang^*^, Rachel Robinson, Richard Vandenberg, Marina Milovic, Lisa Neville

Geological Survey of Canada, 3303-33 Street, NW, Calgary, Alberta, T2L 2A7, Canada

*Corresponding author: Dennis.jiang@nrcan-rncan.gc.ca

**Supplemental Materials:**

**Table S1**. Rock-Eval analytical results on sediment samples from Romulus Lake and Twin Lakes after being prepared in a non-cleaned vacuum-freeze-drier at -80 °C (freeze-dried) *vs* being dried by standing in a fume hood at ambient conditions (air-dried).

Note: S1 and S2 are mg HC/g sediment; S3: mg CO2/g sediment; S3CO: mg CO/g sediment; Tmax: peak temperature of S2 peaks; TOC content: total organic carbon; MinC conttent: mineral carbon; HI: hydrogen index; OI: oxygen index; PC: productive organic carbon; RC: residual organic carbon; PI: production index.


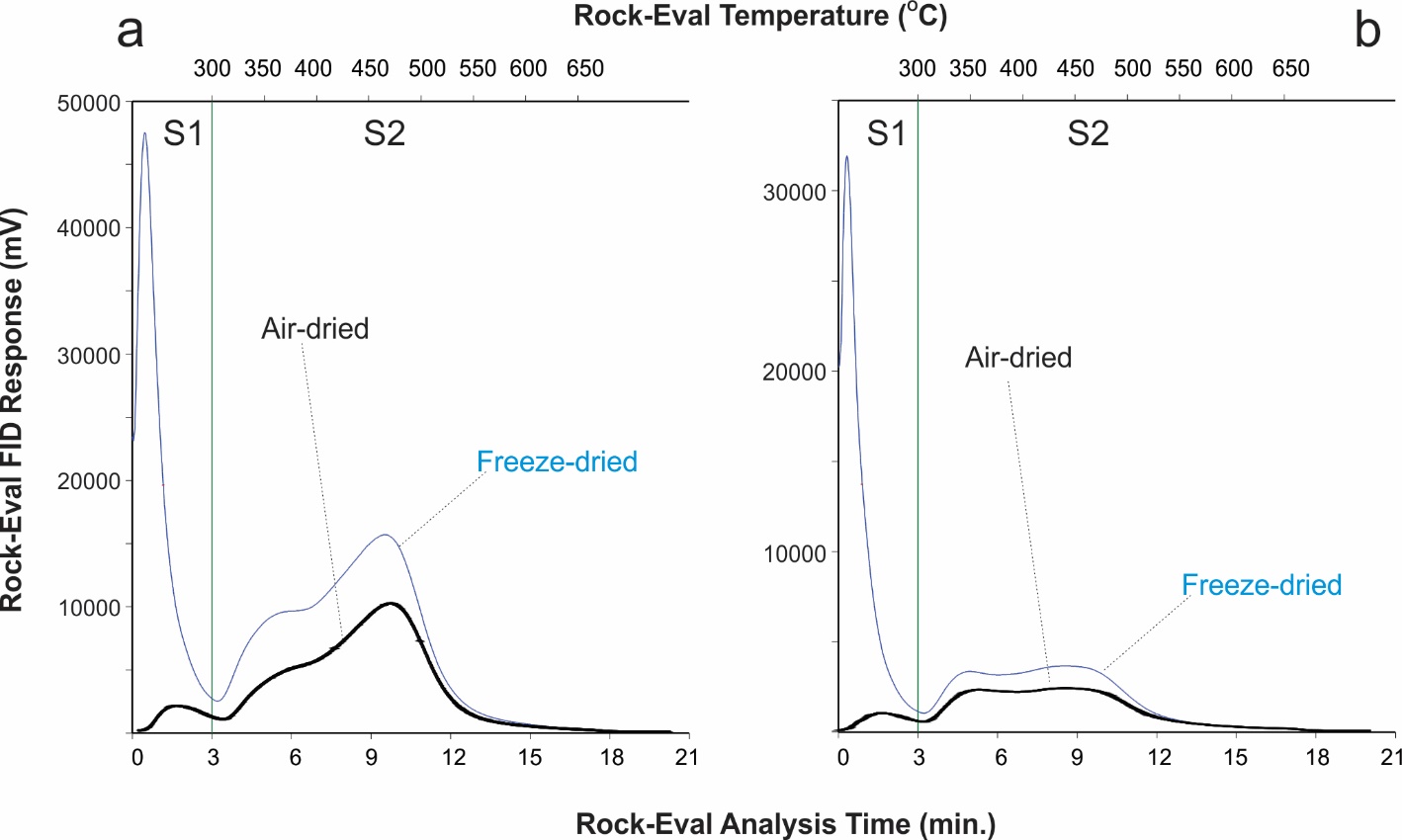


**Figure S1**. FID hydrocarbon pyrograms from Rock-Eval pyrolysis of freeze-dried (thin blue line) prepared in a non-cleaned freeze-drier *vs* air-dried in fume hood at ambient conditions (thick black line) sediment samples from (a) Romulus Lake; and (b) Twin Lakes in the Canadian High Arctic. See Table S1 for the related Rock-Eval parameters.


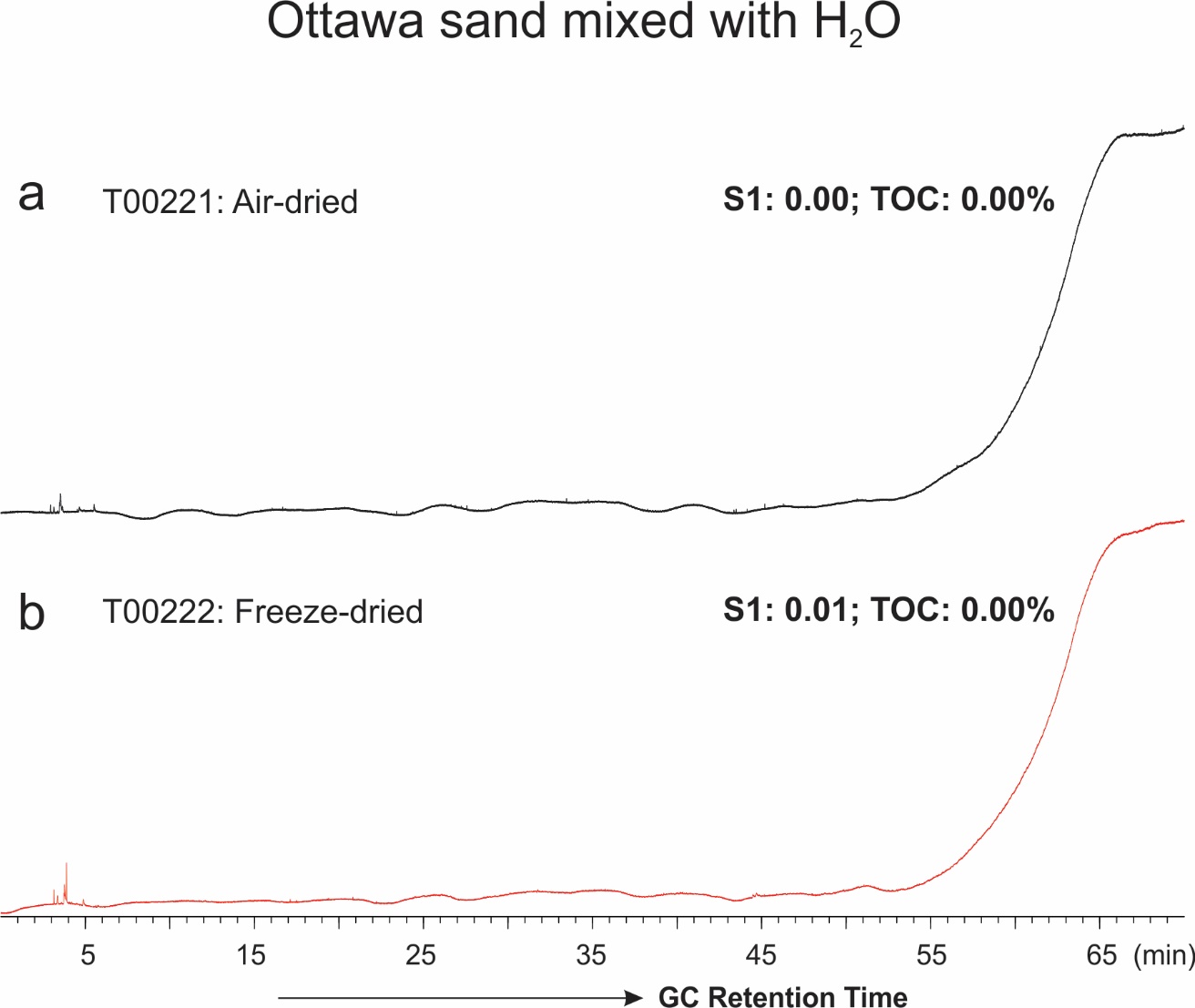


Figure S2. TD-GC traces showing the molecular compositions of Rock-Eval S1 equivalent volatile and semi-volatile hydrocarbons in the (a) air-dried *vs* (b) freeze-dried Ottawa sand samples. Note that GC response has been normalized to the weight of samples. No oil contamination was found occurring the sand sample during freeze-drying.


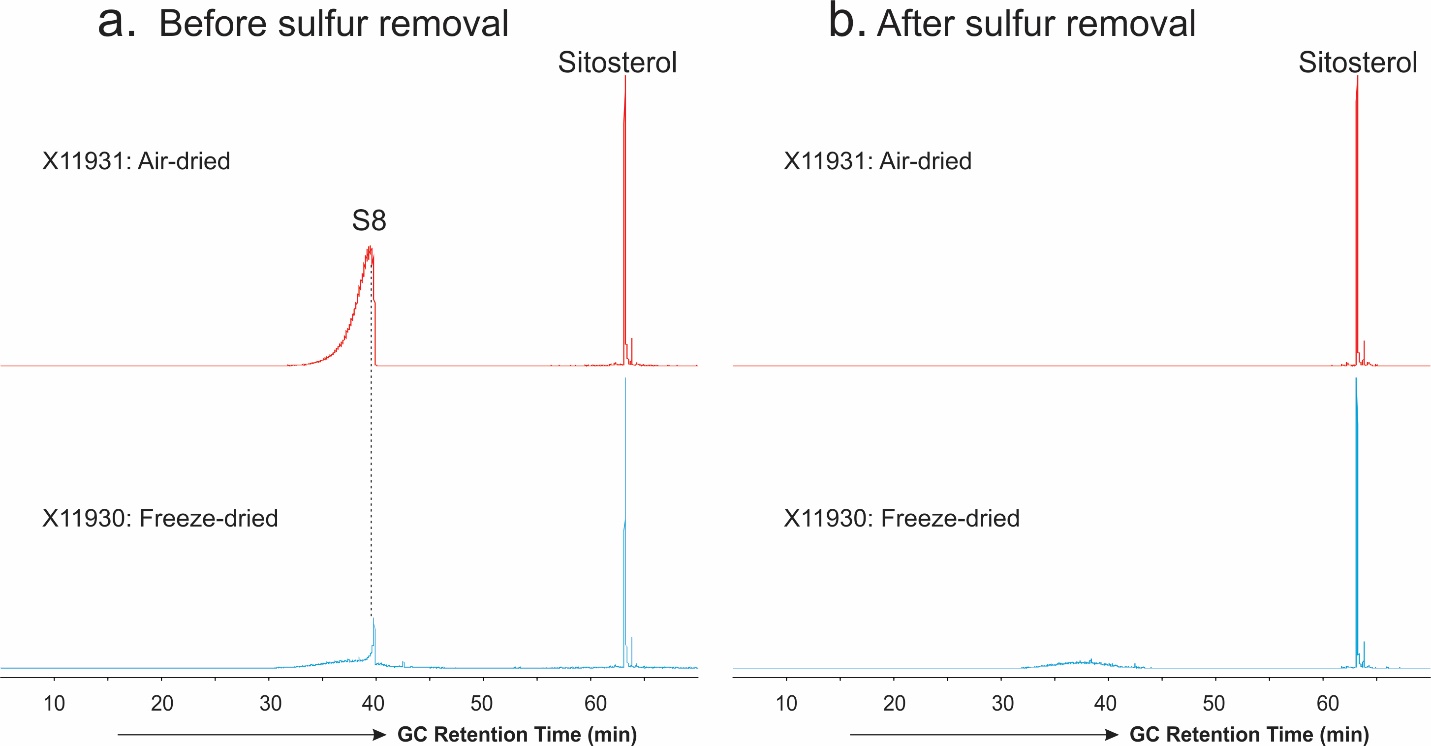


Figure S3. Mass chromatograms m/z (256 + 414) showing the distributions of elemental sulfur (S8) relative to the β-sitosterol (tentatively identified) in solvent extracts from freeze-dried (blue) *vs* air-dried (red) Romulus Lake sediment samples (a) before and (b) after removal of elemental sulfur using fresh copper debris. Increased amount of elemental sulfur was detected in the air-dried than in the freeze-dried sample, likely indicating occurrence of more intense oxidation of sulphides during air-drying than freeze-drying. The elemental sulfur extracted by the solvent in both samples were completely removed by mixing with excess fresh copper. The 30-45 min UCM hump in the freeze-dried sample is due to oil contamination as discussed in the paper.
